# Effect of a Phytogenic Water Additive on Growth Performance, Blood Metabolites and Gene Expression of Amino Acid Transporters in Nursery Pigs Fed with Low-Protein/High-Carbohydrate Diets

**DOI:** 10.3390/ani11020555

**Published:** 2021-02-20

**Authors:** Cedrick N. Shili, Mohammad Habibi, Julia Sutton, Jessie Barnes, Jacob Burch-Konda, Adel Pezeshki

**Affiliations:** Department of Animal and Food Sciences, Oklahoma State University, Stillwater, OK 74078, USA; cedrick.shili@okstate.edu (C.N.S.); mohammad.habibi@okstate.edu (M.H.); julia.sutton@okstate.edu (J.S.); jessie.barnes@okstate.edu (J.B.); burchko@okstate.edu (J.B.-K.)

**Keywords:** low-protein diets, phytogenic additive, growth performance, nutrient digestibility, metabolites, amino acids, amino acid transporters

## Abstract

**Simple Summary:**

Low-protein (LP) diets can be potentially used to reduce the excretion of nitrogenous compounds and feed cost in commercial swine production; however, new strategies are required to be developed to improve the growth performance of pigs receiving these diets. Little is known about the effect of phytogenic additives on the performance of pigs fed with LP diets and the underlying factors involved. The objective of this study was to assess the effect of a phytogenic water additive (PWA) on growth performance, nutrient digestibility, blood metabolites, plasma amino acid (AA) concentration, and gut and skeletal muscle AA transporters in nursery pigs fed with LP diets. Supplemental PWA increased the concentration of circulating essential AA, reduced the transcript of some of the AA transporters in the small intestine and skeletal muscle, improved growth performance when the dietary protein was adequate, and increased muscle lean%, but reduced muscle fat% when the dietary protein was deficient. The used PWA in this study had differential effects on blood calcium and its digestibility depending on the level of dietary protein. This study suggests that PWA can be used for improving the meat composition in protein-restricted pigs, but PWA improves growth performance only when dietary protein is adequate.

**Abstract:**

The objective of this study was to investigate the effect of a phytogenic water additive (PWA) on growth performance and underlying factors involved in pigs fed with low-protein (LP)/high-carbohydrate diets. Forty-eight weaned barrows were allotted to six treatments for 4 weeks: CON-NS, control (CON) diet-no PWA; CON-LS, CON diet-low dose PWA (4 mL/L); CON-HS, CON diet-high dose PWA (8 mL/L); LP-NS, LP diet-no PWA; LP-LS, LP diet-low dose PWA; LP-HS, LP diet-high dose PWA. Relative to CON-NS, pigs fed with CON-HS had increased average daily gain, body weight and serum calcium (Ca) and phosphorous (P) and had decreased mRNA abundance of solute carrier family 7 member 11 and solute carrier family 6 member 19 in jejunum. Compared to LP-NS, pigs fed with LP-HS had increased muscle lean%, decreased muscle fat%, decreased serum Ca and increased serum P. Compared to their NS counterparts, CON-LS, CON-HS, and LP-LS increased the concentration of plasma essential AA and those fed with CON-HS and LP-HS tended to reduce the abundance of the solute carrier family 7 member 1 transcript in skeletal muscle. Thus, PWA improved the performance of weaned pigs fed with protein-adequate diets likely through increased blood essential AA and affected the muscle composition when dietary protein was deficient.

## 1. Introduction

The intensive swine production in developed countries has raised environmental and sustainable agriculture concerns associated with the excretion of large amounts of waste including nitrogen (N) or nitrogen-containing compounds [1,2]. Therefore, new strategies need to be developed to reduce N excretion from the swine industry to the environment. Low-protein (LP) diets are used to reduce N excretion and other toxic nitrogenous compounds in the swine industry [3,4,5]. These diets have also been reported to reduce feed cost and alleviate the incidence of diarrhea in early weaning pigs [6,7]. A slight reduction in dietary crude protein (CP), i.e., <25%, along with supplementing limiting amino acids (AA, i.e., lysine, methionine, tryptophan, and threonine) do not appear to have a negative impact on the performance of pigs [3,5,8,9]. However, in order to justify the use of LP diets at larger scales in commercial swine production, additional research is needed to further improve the growth performance of pigs fed with LP diets.

Phytogenic additives are substances originated from plants. These compounds have received increasing attention lately for use in swine and poultry production due to their positive effects on animal production and health [10,11,12,13,14,15]. Improvement in growth performance was observed when adequate-protein diets were supplemented with phytogenic additives such as Chinese herbal powder in early weaned piglets [16] and herb extract mixture (buckwheat, thyme, curcuma, black pepper, and ginger) in growing pigs [17]. Little is known about whether supplemental phytogenic additives have beneficial effects on the growth performance of pigs fed with LP diets. Manzanilla et al. (2009) showed that supplementing a plant extract mixture to a LP diet (18% CP) in weaned pigs had differential positive or negative effects on various variables measured [18]. In turn, Abousekken et al. (2015) reported that adding *Moringa oleifera* leaf extract to drinking water of broilers fed with LP diet (100 mL/L water) improved growth performance parameters such as weight gain and feed conversion ratio [19]. However, others showed that supplementing a plant extract YGF251 to a diet with very low protein content (14% and 12.5% CP) did not affect the growth performance of growing pigs [20]. Given the very low protein content of the diet used in the former study by Lei et al. (2019) [20] and the marginal effects of feed additives, it appears that the beneficial effects of the plant extract used were not fully explored in that study.

The improved performance in pigs fed with standard protein diets supplemented with phytogenic additives has been linked with improved feed intake and palatability [17,21], immune function [22,23] and blood total antioxidant capacity [24]. Further, phytogenic additives have been reported to enhance intestinal development, ecosystem and microbiota, and nutrient digestibility in pigs fed with standard protein diets [17,23,24,25,26,27]. Data on the mechanisms by which phytogenic additives may influence the growth of pigs fed with LP diets are scarce. Little is known about the effect of phytogenic additives on nutrient digestibility when pigs are offered LP diets. This is particularly important as LP diets have been shown to induce a negative effect on the digestibility of nutrients such as phosphorous (P) in monogastrics [28,29]. Xue et al. (2017) showed that ileal-digested P was decreased when the dietary protein content was reduced in growing pigs [28]. In a different study, Xue et al. (2016) reported a reduced total tract retention of P in broiler chickens fed with diets with low protein content [29]. Further, we previously showed that the concentrations of important blood essential AA such as isoleucine, valine, phenylalanine, tyrosine and tryptophan were decreased in pigs fed with LP diets [30,31], which can influence animal health and growth performance.

There is evidence that nutrient composition can alter intestinal AA transporter expression in pigs fed with LP diets [32]. Reyer et al. (2017) reported that incubating jejunal cells with a combination of essential oils and saponins at medium and high doses stimulated solute carrier family 15 member 1 (SLC15A1) recruitment to the cytoplasmic membrane in growing broilers [33]. Little is understood about whether phytogenic additives can change the concentration of blood AA as well as the expression of AA transporters in the gut and skeletal muscle of pigs fed with LP diets. We hypothesized that supplementing LP diets with a phytogenic additive would improve the growth performance of pigs through alterations in nutrient digestibility, blood metabolites and AA profile, the expression of intestinal and skeletal muscle AA transporters and blood antioxidant capacity. Therefore, the objective of this study was to investigate the effect of a phytogenic water additive (PWA) on growth performance, nutrient digestibility, blood metabolites, AA profile and total antioxidant capacity, and gene expression of AA transporters in the gut and skeletal muscle of nursery pigs fed with low-protein/high-carbohydrate diets.

## 2. Materials and Methods

### 2.1. Animals, Housing, and Diets

All experimental procedures were performed in accordance with the Oklahoma State University Animal Care and Use Committee and were approved by this committee (Animal Care and Use Protocol # AG-18-6). Forty-eight weanling (three weeks old; 6.3 ± 1.2 kg body weight) crossbred barrows (Duroc sire line and Large White X Landrace dam) were used in this study (Seaboard, Hennessey, OK, USA). Upon arrival, pigs were group-housed and acclimated to the environment in a facility with controlled temperature, lighting, and ventilation. The animal facility’s temperature was set at 31 °C in the first week and then was reduced weekly by 1 °C.

Following 2 weeks of adaptation, all pigs (total *n* = 48) were weight matched (9.02 ± 0.17 kg), individually housed, and randomly allotted to one of the 6 dietary treatments (*n* = 8/treatment) in a completely randomized design with 3 × 2 factorial arrangements with the factors of PWA supplement (0, 4 and 8 mL/L of water) and dietary protein content (control and LP) for 4 weeks. Treatments included (1) control (CON) diet with no PWA supplement (CON-NS), (2) CON diet with a low dose of PWA supplement (4 mL/L of water) (CON-LS), (3) CON diet with a high dose of PWA supplement (8 mL/L of water) (CON-HS), (4) LP diet with no PWA supplement (LP-NS), (5) LP diet with a low dose of PWA supplement (4 mL/L of water) (LP-LS) and (6) LP diet with a high dose of PWA supplement (8 mL/L of water) (LP-HS). The used doses for PWA (Herbanimals^®^, Oklahoma City, OK, USA) were based on guidelines provided by Herbanimals Supplement LLC (Oklahoma City, OK, USA) and the route of administration was based on previous study [34]. The ingredients of PWA (Herbanimals^®^) used in the current study include 4.83% *Pandanus amaryllifolius Roxb*, 24.15% *Phyllanthus niruri*, 4.83% *Amomum cardamomum*, 13.04% *Zingiber zerumbet*, 14.49% *Apium Graveolens*, 14.49% *Anethum Graveolens*, 4.83% *Ocimum americanum*, 4.83% *Cinnamomum burmannii Blume*, 4.83% *Myristica fragrans Houtt* and 9.66% *Zingiber officinale roscoe*. The ingredients and analyzed composition of Herbanimals^®^ for vitamins and minerals are given in Table S1.

Phase feeding was applied according to the recommendations of the nutrient requirement of swine [35]. Nursery phase 1 (N1) diet was fed for 1 week (days 1–7 of study), nursery phase 2 (N2) diet was fed for 2 weeks (days 8–21 of study) and nursery phase 3 (N3) diet was offered for 3 weeks (days 22–42 of study) (Table 1). To achieve the desired CP levels and maintain the energy content consistent between the CON and LP diets, the amount of soybean meal as the primary source of protein was decreased, and corn as the major source of carbohydrate was increased in LP diets. Therefore, LP diets, as expected, had a higher carbohydrate content than CON diets (Table 1). LP diets were supplemented with limiting AA (i.e., lysine, methionine, threonine, and tryptophan) at levels equal to the CON diet. All diets contained 0.5% chromium oxide as an indigestible marker for apparent total tract digestibility tests. All pigs had free access to feed and water throughout this study.

### 2.2. Growth Performance 

The individual feed and water intakes were recorded daily and body weight was measured weekly. The average daily gain (ADG), average daily feed intake (ADFI), cumulative feed intake (CFI), weight gain to feed intake ratio (G:F), and weight gain to protein intake ratio (G:P) were computed by using the individual feed intake and body weight records and analyzed dietary protein concentration. The weekly body weight gain (BWG), cumulative feed intake (CFI), cumulative protein intake (CPI), and G:F and G:P ratios were calculated for 4 weeks of data collected.

### 2.3. Feed and Fecal Samples Collection

Approximately 500 g feed samples were collected after mixing each diet and stored at −20 °C until analysis. Fecal samples were collected at week 6 of this study following transferring the pigs to metabolic crates for 24 h, where they had free access to feed and water. Following collection, the fecal samples were stored at −20 °C until analysis. 

### 2.4. Blood and Tissue Samples Collection

At the end of this study, blood samples were drawn from the jugular vein of all pigs in the supine position in 10 mL sterile dry and 3 mL sterile sodium heparin-coated tubes (BD, Franklin Lakes, NJ, USA) for collection of serum and plasma, respectively. Blood samples were placed on ice after collection, transferred to the laboratory, and centrifuged at 4 °C for 10 min and at 2000× *g* to collect the serum or plasma. The collected serum and plasma were stored at −80 °C until further analysis. After blood collection, all pigs were euthanized via CO_2_ asphyxiation, and skeletal muscle (i.e., Biceps femoris) and jejunum samples were collected. Tissue samples were rinsed with distilled water after dissection, snap-frozen in liquid nitrogen, and stored at −80 °C until later processing. Separate muscle samples were collected (~150 g) and stored at −20 °C for lean and fat content analysis.

### 2.5. Feed and Fecal Samples Composition Analysis

As we previously described [36], feed samples were analyzed by ServiTech Laboratories (Dodge City, KS, USA) for dry matter, crude fiber, calcium (Ca), P, N, and chromium using official methods of analysis of AOAC [37]. Fecal samples were analyzed by ServiTech Laboratories (Dodge City, KS, USA) for Ca, P, N, and chromium using the methods indicated above for analysis of feed samples.

### 2.6. Plasma Nitrogen-Containing Compounds Analysis 

Plasma samples were analyzed for nitrogen-containing compounds using a LI-BASED Hitachi 8900 (Hitachi High-Technologies Corporation, Tokyo, Japan) as previously described [38] at Molecular Structure Facility, Proteomics Core (UC Davis Genome Center, Davis, CA, USA). Briefly, plasma samples were thawed at room temperature, acidified to 2% sulfosalicylic acid and incubated for 15 min at room temperature. Samples were then stored at −20 °C overnight. The following day, the samples were thawed and diluted with Li sample diluent (Pickering Labs, Mountain View, CA, USA) containing 100 nmol/mL AE-Cys, before injection (50 μL). The ion-exchange chromatography using a HITACHI L-8900 Amino Acid Analyzer (Hitachi High-Technologies Corporation, Tokyo, Japan) with a post-column ninhydrin reaction was used to separate free AA. Column and buffers were provided by Hitachi (Tarrytown, NY, USA), and ninhydrin was provided by Wako Chemicals (Richmond, CA, USA). The AA standards (Sigma-Aldrich, St. Louis, MO, USA) were used for the calibration of the Amino Acid Analyzer. Absorbances were measured at both 570 and 440 nm after the reaction with ninhydrin to regulate the response factor for each AA and to quantify AA concentrations relative to the known AA and related compound standards (0.5 μmole/mL in 0.2 N lithium citrate, pH 2.2 containing 0.1% phenol and 2% thiodiglycol). The internal standard (AE-Cys, Sigma #A2636) was included due to variations in injection volume that might be caused by the autosampler.

### 2.7. Muscle Composition Analysis 

Muscle samples collected from each pig were scanned by dual-energy X-ray absorptiometry (DEXA; Hologic, Discovery QDR Series, Bedford, MA, USA) to determine the lean and fat content using the rodent’s calibration feature. 

### 2.8. Serum Metabolites Analysis

The concentrations of Ca, P, alkaline phosphatase (ALP), and blood urea nitrogen (BUN) were measured in serum samples (>300 µL) using an automated chemistry analyzer system (CLC 480/BioLis24i, Carolina Liquid Chemistries Corp., Brea, CA, USA). After calibration of the equipment (Catalogue # BL-442600, Multi-Analyte calibrator for Synchron CX/LX), the reagents of Ca (Product #BL251), P (Product #BL218), ALP (Product #BL206), and BUN (Product #BL252) were used to quantify the concentration of these parameters in serum. The absorbances of Ca and P were recorded at 660 and 340 nm, respectively. The absorbances of BUN and ALP were measured at 405 nm.

### 2.9. RNA Isolation and RT-qPCR Analysis

Total RNA extraction, reverse transcription, and quantitative PCR were performed as we described previously [39]. Approximately 300 mg of skeletal muscle and jejunum tissues were grounded using a mortar and pestle and liquid nitrogen. The ground samples then were homogenized with 1 mL Qiazol (Qiagen, Catalogue #74106, Germantown, MD, USA). The RNA was isolated using RNeasy^®^ mini kit (Qiagen, Catalogue #74106, Germantown, MD, USA). The isolated RNA concentration was quantified and the ratio of absorbance at 260 nm and 280 nm was recorded using a Nanodrop ND-1000 spectrophotometer (Thermo Fisher, Waltham, MA, USA). The 260/280 nm absorption ratios for all RNA samples were 1.7–2.0. RNA samples were DNAse treated (Thermo Fisher, Waltham, MA, USA) and used for complementary DNA (cDNA) synthesis. cDNA was synthesized in a 20 μL reaction volume using 5 μL of DNase-treated RNA (1.25 μg), 4 μL of 5× first-strand buffer, 2 μL of random primers (3 μg/μL), 2 μL of deoxyribonucleotide triphosphate (dNTP) mix [2.5 mM], 2 μL of dithiothreitol (DTT) (0.1 M), 1 μL of RNaseOUT (40 U/μL), 1 μL of superscript II reverse transcriptase (200 U/μL), and 3 μL of RNase/DNase-free water (Thermo Fisher Scientific Waltham, MA) using the following program: 22 °C for 5 min, 42 °C for 30 min, 85 °C for 5 min, and terminated at 4 °C in a thermocycler (T100^TM^ Thermal Cycler, Bio-Rad, Hercules, CA, USA). The primers’ sequences were obtained from previous publications as listed in Table 2 [40,41,42,43]. Using a 96-well plate, the real-time quantitative PCR (qPCR) was performed using a CFX96 real-time PCR detection system (Bio-Rad, Hercules, CA, USA). The PCR was performed in a 25 μL total volume reaction, with 2 μL of the first-strand cDNA, 12.5 μL of SYBR Green master mix (Applied Biosystems Inc., Waltham, MA, USA), 0.2 μL of F, 0.2 μL of R sequences of each primer (100 mM), and 10.1 μL of RNase/DNase-free water (Thermo Fisher Scientific, Waltham, MA, USA). The qPCR program used was: denaturation at 50 °C for 2 min and 95 °C for 10 min, 40 cycles amplification at 95 °C for 15 s and 60 °C for 1 min; then a melt curve program: 95 °C for 15 s, 60 °C for 1 min, and 95 °C for 15 s. Finally, the 2^−∆∆CT^ method [44] was used for the calculation of the mRNA abundance of target genes that were normalized to β-actin mRNA abundance as a housekeeping gene. 

### 2.10. Total Antioxidant Capacity

Total antioxidant capacity (TAC) of serum was analyzed using a TAC kit (ab65329, Abcam, Cambridge, MA, USA) following the manufacturer’s instructions. Briefly, after preparing all the reagents to work concentrations, 100 μL of standards solution, 5 μL of serum samples, 5 μL of protein masks, and 90 μL of water were added to each well of a 96-well plate in duplicate. The plate was placed on a plate shaker in the dark for 1.5 h at room temperature to reduce the added 100 μL of Cu^2+^. After the reduction of Cu^2+^, the solution was chelated with a colorimetric probe, and absorbance was measured at 570 nm using an Epoch microplate reader spectrophotometer (BioTek^®^ instrument, Inc., Winooski, VT, USA). The intra-assay coefficient of variation (CV) was 6.55%.

### 2.11. Apparent Fecal Digestibility of Nutrients 

As we previously described [36], apparent fecal digestibility (AFD) of Ca, P, and N was calculated using the index method with chromium as an external marker. The following equation was used to calculate the AFD of nutrients: 100 − (100 × (marker concentration in feed/marker concentration in feces) × (nutrient concentration in feces/nutrient concentration in feed). 

### 2.12. Statistical Analysis 

As we previously described [36] using Al-Therapy Statistics (https://www.ai-therapy.com/psychology-statistics/sample-size-calculator (accessed on 3 July 2018)) and data from our previous study [30], we measured the sample size before starting this study. Our power analysis showed that when 8 pigs/dietary group is used, a 7.90 kg difference in body weight between two groups (SD: control diet = 4.27 kg, low-protein diet = 4.26 kg) can be detected with 93% power (α = 0.05; effect size = 1.852). The ADG, ADFI, final body weight, average daily water intake (ADWI), average daily protein intake (ADPI), G:F and G:P ratios, AFD of nutrients, blood metabolites, DEXA, TAC, and gene expression data were analyzed using two-way ANOVA including the main effects of protein, PWA and protein × PWA in the model (SPSS^®^, IMB SPSS Statistics version 23, Armonk, NY, USA). The difference between the means of treatments for all parameters was separated using pairwise Student’s *t*-test corrected by the Benjamini–Hochberg procedure [45] with 0.1 false discovery for 5 comparisons, i.e., CON-NS vs. CON-LS, CON-NS vs. CON-HS, CON-NS vs. LP-NS, LP-NS vs. LP-LS, LP-NS vs. LP-HS. The daily feed intake, weekly body weight, and weekly growth performance data were analyzed with a linear mixed model of SPSS^®^ (IMB SPSS Statistics version 23, Armonk, NY, USA), with the effect of protein, PWA, time, protein × PWA, protein × time, PWA × time and protein × PWA × time as fixed and the pig as a random variable included in the model. Based on the smallest values of fit statistics for the corrected Akaike Information Criterion (AIC) and the Bayesian Information Criterion (BIC), the covariance structure of the repeated measurements model for daily feed intake, and weekly body weight and CPI were autoregressive and for weekly BWG, CFI, CWI, G:F and G:P were heterogeneous autoregressive. *p* ≤ 0.05 and 0.05 < *p* ≤ 0.1 were considered to declare significant difference and trends, respectively.

## 3. Results

### 3.1. Growth Performance 

The initial BW was not different among groups (9.02 ± 0.17 kg; Table 3). There was a significant effect of dietary protein (*p* < 0.05) on final BW and ADG, with LP pigs having lower final BW (24.37 vs. 26.83 kg) and ADG (0.55 vs. 0.63 kg) than CON pigs (Table 3). However, there were no differences between LP-NS and CON-NS pigs on final BW and ADG. The effect of protein × PWA on final BW and ADG tended to be significant (*p* < 0.1; Table 3). While there were no differences in ADG and final BW of pigs fed with LP-NS, LP-LS, and LP-HS, pigs in CON-HS had 19% higher ADG and tended to have 15% higher final BW compared to those fed CON-NS (Table 3; Figure S1A). When the data were analyzed on a weekly basis, there was a significant protein effect on BWG, with LP pigs having lower BWG than CON counterparts during the first (3.60 vs. 4.10 kg) and second week (3.53 vs. 4.13 kg) of the study (*p* < 0.01; Table 4). No differences in BWG were detected among treatments during the first three weeks, but pigs fed CON-HS had 34% higher (*p* < 0.05) BWG than those fed with CON-NS at week 4 (Table 4).

The effects of protein, PWA, and protein × PWA on ADFI and ADWI were not significant (Table 3). There was an overall significant effect of time (*p* < 0.01) on FI, with pigs fed with CON-HS tending to have a 70% higher feed intake compared to those fed with CON-NS on day 5 (Figure S1B). Cumulative feed intake did not change among groups during the entire study (Table 4). When CPI was assessed on a weekly basis, there was a significant effect of protein during all four weeks of this study, with LP pigs having a lower CPI than CON pigs (0.57 vs. 0.88, 0.92 vs. 1.31, 1.26 vs. 1.71, 1.53 vs. 2.11 kg for weeks 1 to 4, respectively). Additionally, the effect of PWA on CPI was significant in the second week of study with a dose-dependent increase in CPI (0.99, 1.42, and 1.22 kg for NS, LS, and HS, respectively). There was a significant effect of protein × PWA on CPI during week 4 of this study, suggesting that PWA improved the CPI only with a standard level of dietary protein (Table 4). During weeks 2 and 3, pigs fed LP-NS tended to have 30% and 28% less CPI than those fed with CON-NS, respectively, while pigs from the CON-HS group tended to have a 20% higher CPI during the second week compared to CON-NS pigs. Additionally, pigs fed LP-HS tended to have a 26% higher CPI than those fed with LN-NS during the second week of this study. Pigs in the LP-LS group had 27% higher ADWI than pigs fed with LP-NS (Table 3). When water intake was analyzed weekly, there was a significant effect of protein on CWI during week 2 of this study with LP pigs having a lower water intake than CON (18.30 vs. 24.13 L) (Table 4). Pigs fed with LP-NS consumed 48% less water during the second week compared to those fed with CON-NS. Pigs fed with LP-HS consumed 83% more water compared to animals fed with LP-NS during the second week of this study. On week 4, LP-LS pigs tended to consume 47% more water than LP-NS. 

There was a significant effect of protein (*p* < 0.05) on G:F and G:P ratios (Table 3). Relative to CON, LP pigs had lower G:F (0.62 vs. 0.70) and greater G:P ratios (3.59 vs. 2.96). However, no differences in G:F ratio were detected when CON-NS and LP-NS pigs were compared. Pigs fed with LP-NS diet had a 29% higher G:P than the CON-NS group. There was a significant protein effect on G:F ratio (*p* < 0.05) on week 1, with LP pigs having a lower G:F ratio compared to CON pigs (0.51 vs. 0.78). Additionally, the effect of PWA on G:F ratio was significant (*p* < 0.05) in week 1 and 2, with a dose-dependent increase on G:F ratio during the first week (0.50, 0.59, and 0.83) and a decrease in G:F ratio during the second week (0.77, 0.76 and 0.62) for NS, LS, and HS, respectively (Table 4). A significant protein effect (*p* < 0.05) was detected on weekly G:P ratio during weeks 2, 3, and 4, with a higher G:P ratio in pigs fed with LP diets than those fed with CON diets (3.88 vs. 3.31, 3.63 vs. 2.94 and 3.60 vs. 3.01, for weeks 2, 3 and 4, respectively). The effect of PWA on the G:P ratio in weeks 1 and 2 was significant. During week 1, increasing the levels of PWA, increased the G:P ratio (2.40, 2.76, and 3.72 for NS, LS, and HS, respectively). Pigs fed with LP-HS had a 57% increase and a 22% decrease in the G:P ratio compared to those fed with LP-NS, during the first and third week of this study, respectively. Pigs fed with LP-NS had a 43% and 32% greater G:P ratio than those fed with CON-NS during the third and fourth weeks of study, respectively (Table 4).

### 3.2. Muscle Fat and Lean Content

Muscle lean% and fat% are shown in Table 3. There was a significant effect of protein, PWA, and protein × PWA (*p* < 0.01) on muscle lean% and fat%. Pigs in the LP group had higher muscle lean% (85.1%) and lower fat% (13.9%) than those in the CON group (81.5% and 17.8% for lean and fat%, respectively). Supplementing diets with PWA dose-dependently increased muscle lean% (81.5%, 82.0%, and 86.6% for NS, LS, and HS, respectively) and decreased muscle fat% (17.9%, 17.1% and 12.7% for NS, LS, and HS, respectively). While there was no difference in muscle lean% and fat% of pigs in CON-LS, CON-HS, and CON-NS, pigs fed with LP-HS had 13% more lean and 55% less fat compared to those fed with LP-NS (Table 3). 

### 3.3. Plasma Nitrogen-Containing Compounds

Plasma nitrogen-containing compounds are shown in Table 5. There was a significant effect of protein (*p* < 0.05) on the majority of plasma AA concentration. Overall, pigs fed LP diets had a lower concentration of plasma leucine, isoleucine, phenylalanine, histidine, arginine, valine, tyrosine, proline, and creatinine, and had higher plasma lysine, methionine, tryptophan, threonine, alanine, and glutamic acids than those fed with CON diets. Pigs fed with LP-NS had lower plasma valine, isoleucine, histidine and 3, methylhistidine, and higher lysine, serine, glycine, sarcosine, and alanine than CON-NS pigs (*p* < 0.05). Overall, pigs in the HS group had a higher concentration of methionine, tryptophan, leucine, isoleucine, valine, phenylalanine, arginine, serine, glutamine, and tyrosine compared to LS and NS groups. Pigs fed CON-HS had a higher plasma concentration of valine, isoleucine, leucine, phenylalanine, tryptophan, lysine, histidine, arginine, serine, glycine, alanine, tyrosine, asparagine, proline, and sarcosine and lower glutamic acid, aspartic acid, and taurine than CON-NS. Moreover, the plasma concentration of leucine, serine, glycine, proline, sarcosine, and alanine was higher, and methionine and lysine tended to be higher in pigs fed with CON-LS than those fed with CON-NS. Pigs fed with LP-LS had a higher (*p* < 0.05) plasma valine, histidine, and alanine and tended to have a higher plasma leucine, isoleucine, phenylalanine, and ornithine compared to those fed with LP-NS. The plasma concentration of histidine, serine, glutamic acid, taurine, and ammonia were lower and lysine and proline tended to be lower in the LP-HS group when compared to LP-NS (*p* < 0.05).

### 3.4. Apparent Fecal Digestibility of Calcium, Phosphorus, and Nitrogen

**Figure 1:**
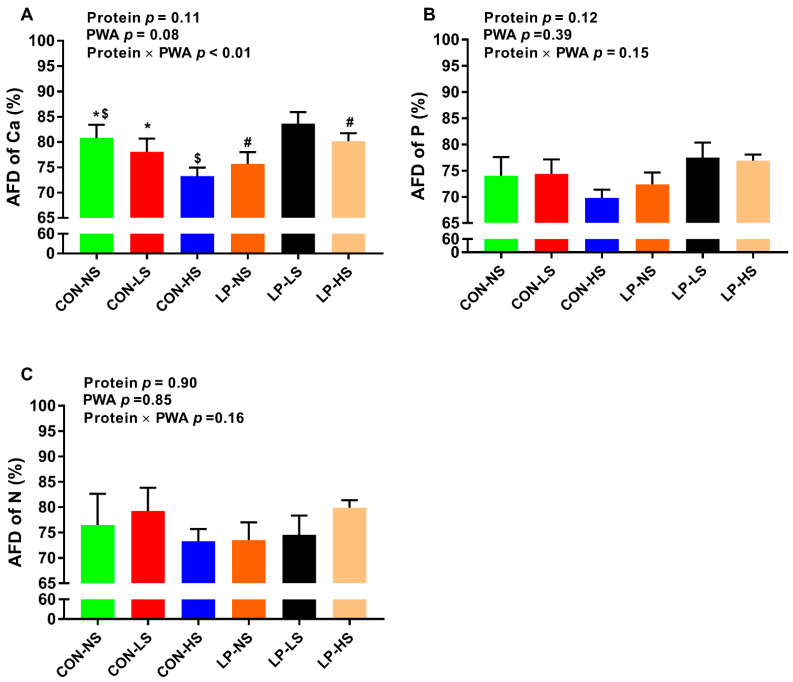
Apparent fecal digestibility (AFD) of (**A**) calcium (Ca), (**B**) phosphorus (P) and (**C**) nitrogen (N) of nursery pigs fed with two levels of dietary protein and three levels of a phytogenic water additive (PWA). CON-NS: control diet with no PWA supplemented, CON-LS: control diet with a low dose of PWA (4 mL/L of water) supplemented, CON-HS: control diet with a high dose of PWA (8 mL/L of water) supplemented, LP-NS: low-protein diet with no PWA added, LP-LS: low-protein diet with a low dose of PWA (4 mL/L of water) added and LP-HS: low-protein diet with a high dose of PWA (8 mL/L of water) added. *^$#^ Among groups, values with a common superscript symbol tend to be different (0.05 < *p* ≤ 0.1). Values are the means ± SEM. *n* = 8/dietary group.

Pigs fed with CON-LS and CON-HS tended to have a lower AFD of Ca compared to those fed with CON-NS (Figure 1A). Additionally, pigs fed with LP-HS tended to have a higher AFD of Ca compared to pigs fed with LP-NS (Figure 1A). There were no differences in AFD of P (Figure 1B) and N (Figure 1C) across dietary groups.

### 3.5. Serum Calcium, Phosphorus, and Alkaline Phosphatase and Blood Urea Nitrogen

**Figure 2:**
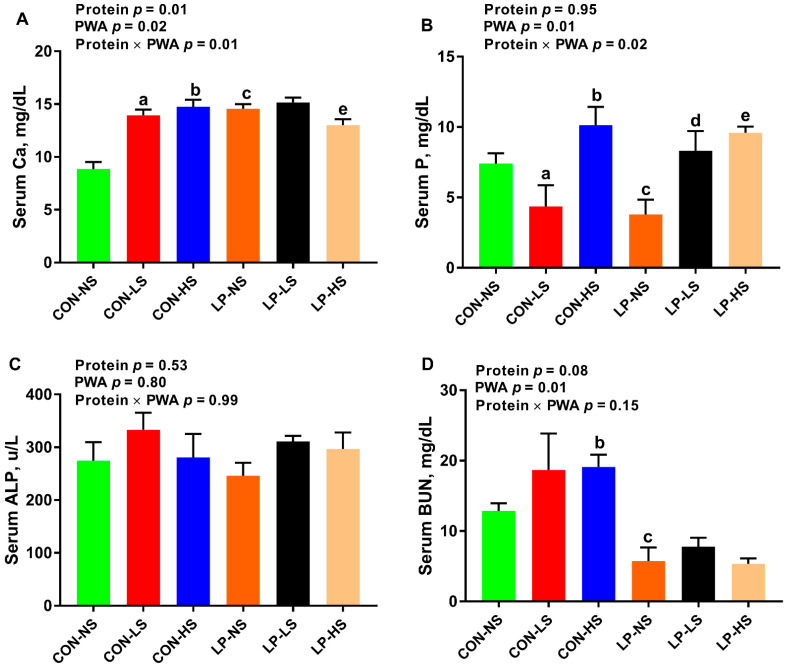
Serum (**A**) calcium (Ca), (**B**) phosphorus (P), (**C**) alkaline phosphatase (ALP) and (**D**) blood urea nitrogen (BUN) of nursery pigs fed with two levels of dietary protein and three levels of a phytogenic water additive (PWA). CON-NS: control diet with no PWA supplemented, CON-LS: control diet with a low dose of PWA (4 mL/L of water) supplemented, CON-HS: control diet with a high dose of PWA (8 mL/L of water) supplemented, LP-NS: low-protein diet with no PWA added, LP-LS: low-protein diet with a low dose of PWA (4 mL/L of water) added and LP-HS: low-protein diet with a high dose of PWA (8 mL/L of water) added. ^a^ *p* ≤ 0.05 CON-NS vs. CON-LS, ^b^ *p* ≤ 0.05 CON-NS vs. CON-HS, ^c^ *p* ≤ 0.05 CON-NS vs. LP-NS, ^d^ *p* ≤ 0.05 LP-NS vs. LP-LS, and ^e^ *p* ≤ 0.05 LP-NS vs. LP-HS. Values are the means ± SEM. *n* = 7–8 for each dietary group.

There was a significant effect of protein, PWA and protein × PWA on serum Ca concentration (*p* < 0.05; Figure 2A). The serum Ca of pigs fed with LP was higher than CON pigs (14.14 vs. 12.50 mg/dL, respectively). The serum Ca concentration for pigs fed with NS, LS and HS were 12.08, 14.57 and 13.30 mg/dL, respectively. Pigs fed with CON-LS, CON-HS and LP-NS had higher serum Ca than animals fed with CON-NS. In addition, LP-HS pigs had a lower serum Ca compared to LP-NS ones (Figure 2A). The effect of PWA and protein × PWA on serum P was significant (*p* < 0.05; Figure 2B). Supplementation of PWA dose-dependently increased the serum P concentration (5.59, 6.03, and 9.72 mg/dL for NS, LS, and HS, respectively). Pigs fed with CON-HS had a higher (*p* < 0.05) serum P compared to those fed with CON-NS, but pigs in CON-LS group had lower serum P compared to those in CON-NS group (Figure 2B). Additionally, pigs fed LP-LS and LP-HS had an increased serum P compared to those fed with LP-NS (*p* < 0.05; Figure 2B). Alkaline phosphatase was used as a marker of bone turnover in the current study. No differences in serum ALP concentration were observed across groups (Figure 2C). The effect of protein on BUN tended to be significant (*p* = 0.08) with a decrease in serum BUN of LP compared to CON pigs (6.78 and 15.75 mg/dL, respectively) (Figure 2D). Pigs fed with CON-HS had a higher serum BUN compared to those fed with CON-NS. Additionally, pigs fed with LP-NS had a lower serum BUN compared to pigs fed with CON-NS (Figure 2D).

### 3.6. The mRNA Abundance of Amino Acid Transporters in the Jejunum and Skeletal Muscle

**Figure 3:**
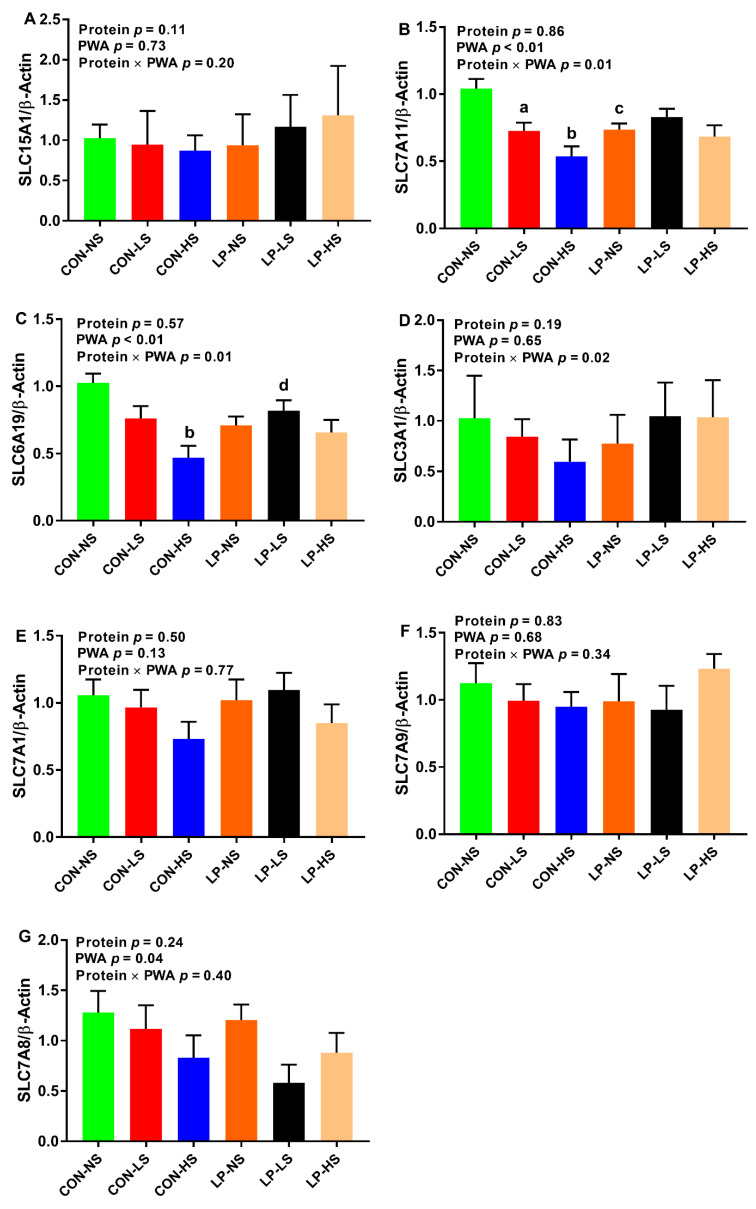
Relative mRNA abundance of (**A**) solute carrier family 15 member 1 (*SLC15A1*), (**B**) solute carrier family 7 member 11 (*SLC7A11*), (**C**) solute carrier family 6 member 19 (*SLC6A19*), (**D**) solute carrier family 3 member 1 (*SLC3A1*), (**E**) solute carrier family 7, member 1 (*SLC7A1*), (**F**) solute carrier family 7 member 9 (*SLC7A9*), and (**G**) solute carrier family 7 member 8 (*SLC7A8*) in the jejunum of nursery pigs fed with two levels of dietary protein and three levels of a phytogenic water additive (PWA). CON-NS: control diet with no PWA supplemented, CON-LS: control diet with a low dose of PWA (4 mL/L of water) supplemented, CON-HS: control diet with a high dose of PWA (8 mL/L of water) supplemented, LP-NS: low-protein diet with no PWA added, LP-LS: low-protein diet with a low dose of PWA (4 mL/L of water) added and LP-HS: low-protein diet with a high dose of PWA (8 mL/L of water) added. ^a^ *p* ≤ 0.05 CON-NS vs. CON-LS, ^b^ *p* ≤ 0.05 CON-NS vs. CON-HS, ^c^ *p* ≤ 0.05 CON-NS vs. LP-NS, ^d^ *p* ≤ 0.05 LP-NS vs. LP-LS. Values are the means ± SEM. *n* = 8 for each dietary group.

The effect of PWA and protein × PWA on mRNA abundance of solute carrier family 7 member 11 (*SLC7A11*) and solute carrier family 6 member 19 (*SLC6A19*) in the jejunum (Figure 3B,C) was significant. The relative mRNA abundance of *SLC7A11* was 0.88, 0.76, and 0.61 for NS, LS, and HS, respectively. Further, the effect of PWA on solute carrier family 7 member 8 (*SLC7A8*) was significant (*p* < 0.05), with the relative mRNA abundance of 0.86, 0.81, and 0.56 for NS, LS, and HS, respectively (Figure 3G). Relative to CON-NS, the abundance of the *SLC7A11* transcript was decreased (*p* < 0.05) in the jejunum of CON-LS, CON-HS, and LP-NS (Figure 3B). Pigs fed with CON-HS had a decreased mRNA abundance of *SLC6A19* compared to those fed with CON-NS (Figure 3C). Pigs fed with LP-LS had an increased transcript of *SLC6A19* compared to the LP-NS group (Figure 3C). No differences were seen among the treatments for the abundance of the transcript of solute carrier family 15 member 1 (*SLC15A1*), solute carrier family 3 member 1 (*SLC3A1*), solute carrier family 7 member 1 (*SLC7A1*), and solute carrier family 7 member 9 (*SLC7A9*) (Figure 3A,D–F) in the jejunum.

**Figure 4:**
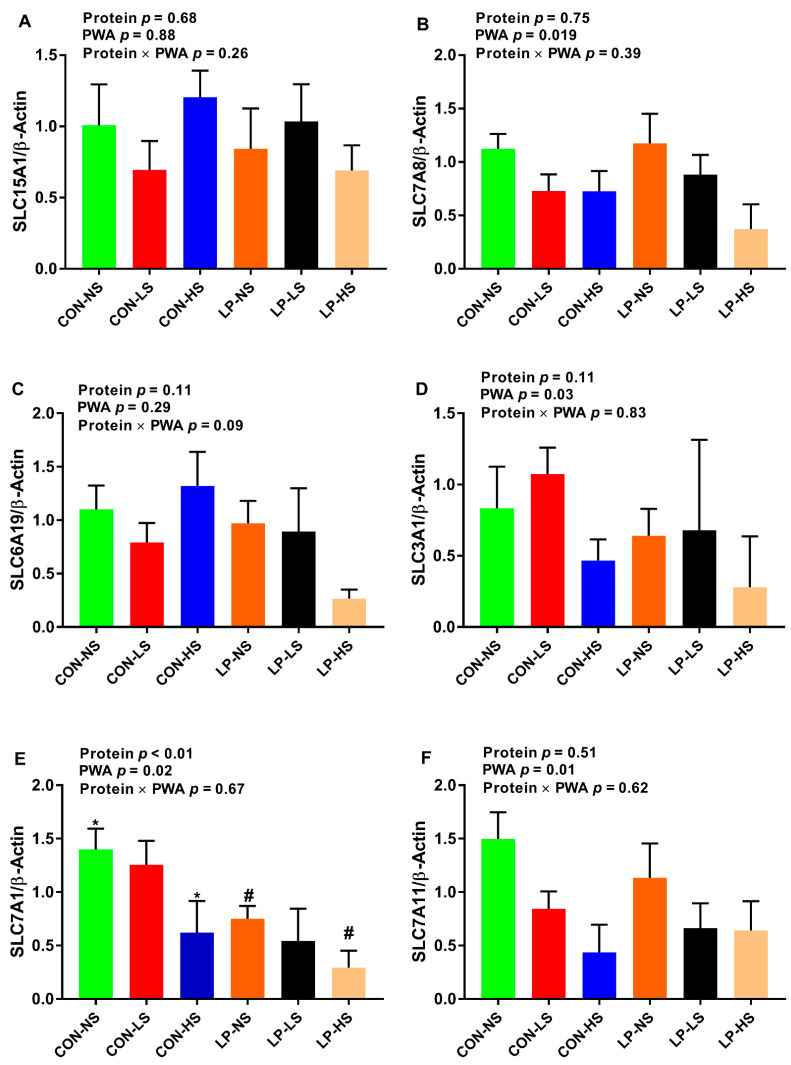
Relative mRNA abundance of (**A**) solute carrier family 15 member 1 (*SLC15A1*), (**B**) solute carrier family 7 member 8 (*SLC7A8*), (**C**) solute carrier family 6 member 19 (*SLC6A19*), (**D**) solute carrier family 3 member 1 (*SLC3A1*), (**E**) solute carrier family 7, member 1 (*SLC7A1*), and (**F**) solute carrier family 7 member 11 (*SLC7A11*) in the skeletal muscle of nursery pigs fed with two levels of dietary protein and three levels of a phytogenic water additive (PWA). CON-NS: control diet with no PWA supplemented, CON-LS: control diet with a low dose of PWA (4 mL/L of water) supplemented, CON-HS: control diet with a high dose of PWA (8 mL/L of water) supplemented, LP-NS: low-protein diet with no PWA added, LP-LS: low-protein diet with a low dose of PWA (4 mL/L of water) added and LP-HS: low-protein diet with a high dose of PWA (8 mL/L of water) added. *^#^ Among groups, values with a common superscript symbol tend to be different (0.05 < *p* ≤ 0.1). Values are the means ± SEM. *n* = 8 for each dietary group.

The effect of PWA on the mRNA abundance of *SLC7A8*, *SLC3A1*, *SLC7A1*, and *SLC7A11* in skeletal muscle was significant (Figure 4B,D–F). The relative mRNA abundance for *SLC7A8* were 1.14, 0.80 and 0.54, for *SLC3A1* were 0.73, 0.87 and 0.37, for *SLC7A1* were 1.07, 0.90, and 0.45 and for *SLC7A11* were 1.32, 0.75 and 0.54 for NS, LS and HS, respectively. Pigs fed with CON-HS tended to have a lower *SLC7A1* gene expression compared to those fed with CON-NS (Figure 4E). Additionally, pigs fed with LP-HS tended to decrease the abundance of the transcript of *SLC7A1* compared to LP-NS (Figure 4E). No differences in the mRNA abundance of *SLC15A1* and *SLC6A19* among dietary treatments were observed in skeletal muscle (Figure 4A,C).

### 3.7. Serum Total Antioxidant Capacity

No differences in serum TAC were observed among dietary treatments, although the effect of protein × PWA on TAC was significant (Figure S2).

## 4. Discussion

The objective of this study was to assess the effect of a PWA on growth performance of nursery pigs fed with low-protein/high-carbohydrate or standard protein diets and the factors involved in this process. Our study revealed several important findings: (1) overall, supplemental PWA increased the ADG and tended to improve BW in CON-HS, but not in LP pigs, suggestive of a positive effect of PWA on growth performance when dietary protein is adequate; (2) regardless of dietary protein content, supplementing PWA increased muscle lean% and decreased muscle fat%, but this effect was significant when PWA was supplemented to LP diets at high dose (LP-HS), but not to CON diets, indicative of a promising effect of PWA on muscle composition when dietary protein is deficient; (3) supplementing CON pigs with a low or high dose of PWA increased the concentration of the majority of plasma essential AA and in LP pigs, a low dose of PWA (LP-LS) recovered the reduced AA levels such as branched-chain AA, phenylalanine, and histidine suggestive of improved digestion and absorption of proteins and AA by PWA; (4) supplementing LP pigs with PWA at high dose (LP-HS) reduced serum Ca concentration while supplementing CON pigs with both low and high doses of PWA increased the serum Ca concentration and decreased the AFD of Ca; (5) CON and LP pigs supplemented with a high dose of PWA had higher serum P concentration; (6) supplementing CON pigs, but not LP pigs, with a high dose of PWA decreased the mRNA abundance of *SLC7A11* and *SLC6A19* in the jejunum and supplementing both CON and LP pigs with a high dose of PWA tended to reduce the abundance of the *SLC7A1* transcript in skeletal muscle. In summary, supplemental PWA improved growth performance when the dietary protein was adequate and the muscle composition when dietary protein was deficient, improved plasma AA profile, and produced differential effects on blood Ca and its digestibility depending on the level of dietary protein.

Little is known about whether supplemental phytogenic additives would improve the growth performance and meat composition of pigs fed with LP diets. Supplementation of PWA improved the ADG and body weight of pigs when diets contained recommended amounts of protein in the current study. Similarly, previous studies have shown a beneficial effect of phytogenic additives on growth performance in early weaned [16] and growing pigs [17] when diets with standard protein levels were fed. In the current study, PWA did not improve the performance of pigs fed with low-protein/high-carbohydrate diets. Likewise, others showed that supplementing very low-protein diets with a plant extract YGF251 did not influence the growth performance of growing pigs [20]. In the present study, the beneficial effects of PWA in pigs fed with CON diet could be due to the increased concentration of blood essential AA that may contribute to improving the growth of pigs, within their genetic potential. Others have reported an enhanced nutrient digestibility in response to supplemental phytogenic additives when pigs are fed with adequate-protein diets [18,24,25,26]. In parallel with our data, an increased serum Ca and other minerals were reported in broiler chicks following herbal extract supplementation [46]. In the current study, the concentration of blood Ca was dramatically increased in experimental diets up to ~15 mg/dL. It has been shown that most animals show systemic hypercalcemia signs when the concentration of Ca is greater than 15 mg/dL [47]. In another study, the concentration of Ca was increased to similar levels seen in our study with no clinical signs of illness when pigs were fed with calcium-replete and P-deficient diets [48]. Similarly, no clinical signs of hypercalcemia were observed in our animals. Hypercalcemia induced renal vasoconstriction, which is characterized by reduced renal blood flow and glomerular filtration rate was shown to occur in dogs with blood Ca concentration higher than 20 mg/dL [47]. Given the subclinical levels of blood Ca and no clinical signs of hypercalcemia in animals in the current study, no failure in kidney function was assumed in our animals. Further, the blood ALP as a marker of bone turnover did not change across dietary groups in the present study. Additional research will be needed to determine the effect of PWA on bone parameters such as bone ash, mineral contents and density. The reduced AFD of Ca in CON pigs supplemented with PWA is likely due to a negative feedback loop that inhibits the release of parathyroid hormone and vitamin D3 and Ca absorption when blood Ca is rising. Little is known about the effect of phytogenic additives on nutrient digestibility when pigs are offered LP diets. We observed a differential response of PWA on blood Ca and P concentration in the current study. Further research is warranted to elucidate the interaction of the dietary protein with PWA on nutrient digestibility. The data on the effect of PWA on muscle composition and its interaction with dietary protein are scarce. Our study showed that regardless of dietary protein content, supplementing PWA increased the lean% and decreased the fat% in the muscle in a dose-dependent manner but this effect was significant when PWA was supplemented to low-protein/high-carbohydrate diet at a high dose. In a recent study, treating porcine primary muscle cell lines with natural phytogenic compounds from fruit peel increased the expression of genes involved in muscle development, and in live animals, these compounds decreased back fat thickness [49]. The data from this study provide evidence that a novel PWA could be used to improve the growth performance and meat composition of nursery pigs. Whether or not the used PWA in the current study could potentially alter back fat thickness or the whole body composition of the carcass, additional research is required to answer that question. While our data show improvement in blood AA profile and increased Ca and P concentration following PWA supplementation, the mode of action of the PWA used in the present study on growth performance and muscle composition has not been fully explored and further studies are needed to better understand the pathways involved.

The positive effects of phytogenic additives on the growth performance of pigs have been linked with the beneficial effects of these additives on eating behavior [17,21], immune function [22,23], antioxidant capacity [24], and intestinal development, ecosystem, and nutrient digestibility [18,24,25,26]. However, less attention has been paid towards the changes in circulating AA while blood AA profile is highly associated with animal health and growth [50]. In the current study, regardless of the level of dietary protein, supplementing PWA improved the plasma AA concentration. More specifically, supplementing CON pigs with a low or high dose of PWA and LP pigs with a low dose of PWA improved the majority of plasma essential AA concentration. Similarly, others reported an increase in serum AA concentration when a phytogenic additive was added to a diet of weaned pigs fed with standard protein diets [51]. In support of data obtained in the present study, an increase in ileal AA digestibility was reported in weaned pigs supplemented with herbal extract, and that improvement was attributed to the positive effect of phytogenic additives on enhancing the functions of digestive enzymes in the gut [14,52]. The improved performance of pigs fed with an adequate-protein diet supplemented with PWA in the current study might be due to the increased concentration of essential AA in blood. Due to the complexity of the composition of herbal extracts containing a wide variety of compounds such as flavonoids, fatty acids, and protein, it remains to be determined whether a group of active compounds or a single chemical moiety of phytogenic additives is stimulating the increased blood AA concentration. 

Amino acids are sensed and transported by specific AA transporters [53,54]. It is known that nutrient composition can alter intestinal AA transporter expression in pigs fed protein-deficient diets [32], but little is known about interactive effects of dietary protein and phytogenic additives on dynamics of these transporters in the gut and skeletal muscle. Supplementing CON pigs with a high dose of PWA decreased the abundance of the *SLC7A11* and *SLC6A19* transcripts in the jejunum and supplementing both CON and LP pigs with a high dose of PWA reduced the abundance of the *SLC7A1* transcript in skeletal muscle. In contrast, others showed that incubating jejunal cells with the combination of essential oils and saponins at medium and high doses stimulated solute carrier family 15 member 1 (*SLC15A1*) recruitment to the cytoplasmic membrane in growing broilers [33]. These controversial results on the effect of PWA on the expression of AA transporters might be due to the differences in the composition and the dose of PWA used. *SLC7A11* is a cystine/glutamate exchanger [55] and is specifically involved in the cysteine and glutamate transport system. The downregulation of jejunal *SLC7A11* in the CON-HS group is likely contributing to the reduced plasma glutamic acid concentration in this group. *SLC6A19* is the major neutral AA transporter in the small intestine and kidney [56]. Given the increased concentration of neutral AA including alanine, asparagine, glycine, isoleucine, leucine, phenylalanine, serine, proline, threonine, tryptophan, tyrosine, and valine in the CON-HS group, the reduced mRNA abundance of *SLC6A19* in the jejunum is suggestive of a feedback regulatory mechanism in this process. A feedback regulatory mechanism has been described between cycling AA pool and expression of intestinal AA transporters [57]. Although the role of *SLC71* is not very well known in skeletal muscle [58], there is evidence that accounts for arginine uptake [59,60]. The reduced expression of *SLC7A1* in skeletal muscle of CON-HS and LP-HS groups and hence decreased uptake of arginine by skeletal muscle may contribute to the greater concentration of arginine in the circulation for these groups. The role of other basic and acidic transporters in the small intestine that contribute to improved AA profile in pigs supplemented with phytogenic additives requires further investigation. 

## 5. Conclusions

Little research has been conducted to explore the interaction of phytogenic additives and dietary protein on growth performance and related underlying factors in young pigs. Overall, supplemental PWA (8 mL/L) increased the concentration of plasma essential AA and reduced the abundance of the transcript for some of the AA transporters in the small intestine and skeletal muscle, improved growth performance when the dietary protein content was adequate and increased muscle lean%, and reduced muscle fat% when the dietary protein was deficient. Depending on the level of dietary protein, supplementation of PWA had differential effects on plasma Ca concentration and its digestibility. Altogether, although the supplemental PWA used in this study failed to improve the growth performance of nursery pigs fed with LP diets, this additive improved the performance of pigs fed with standard protein diets likely through improved blood AA profile and had beneficial effects on muscle composition when dietary protein was deficient. Further research is warranted to elucidate the mechanisms controlling the interactive effects of dietary protein with phytogenic additives on nutrient digestibility and AA transport system. Since phytogenic additives contain a wide variety of bioactive compounds, further investigation is required to understand what active compound(s) of these additives is mediating their beneficial effects.

## Figures and Tables

**Table 1:** Ingredients and chemical composition of experimental diets (as-fed basis).

		Diets ^1^			
	N1	N2		N3	
		CON	LP	CON	LP
**Ingredients, %**					
Corn, yellow dent ^2^	32.21	45.04	64.72	57.06	73.49
Soybean meal, 47.5% CP ^2^	15.00	36.22	13.94	38.39	19.95
Fish meal, menhaden ^2^	6.00	4.90	4.98	2.07	2.11
Whey, dried ^2^	25.00	5.87	5.97	-	-
Lactose ^2^	7.00	-	-	-	-
Corn starch ^2^	-	5.87	5.97	-	-
Plasma spray-dried ^2^	6.00	-	-	-	-
Soy protein concentrate ^2^	2.20	-	-	-	-
Soybean oil ^2^	4.00	-	-	-	-
Dicalcium phosphate ^2^	0.67	0.76	0.85	0.99	1.15
Limestone ^2^	0.45	0.54	0.67	0.62	0.68
Nursery vitamin premix ^3^	0.05	0.19	0.19	0.18	0.20
Salt ^2^	0.50	0.49	0.50	0.57	0.52
Chromium oxide ^2^	-	0.50	0.50	0.50	0.52
Trace mineral premix ^4^	0.06	0.07	0.06	0.07	0.08
Selplex ^2^	0.05	-	-	-	-
Choline Cl ^2^	0.03	-	-	-	-
Zinc oxide, 72% Zn ^2^	0.35	-	-	-	-
L-Lysine, sulfate ^2^	0.17	-	1.12	-	0.88
DL-Methionine ^2^	0.18	-	0.14	-	0.08
L-Threonine ^2^	0.07	-	0.28	-	0.22
L-Tryptophan ^2^	-	-	0.13	-	0.10
**Calculated Chemical Composition ^5^**					
Dry matter, %	92.34	90.72	90.72	89.66	89.60
ME, Mcal/kg	3.52	3.40	3.40	3.30	3.32
Crude protein, %	22.99	24.71	17.03	24.26	17.83
Crude fiber, %	1.39	2.31	2.01	2.64	2.39
Crude fat, %	6.68	3.36	3.46	3.57	3.66
Nitrogen, %	3.65	3.95	2.72	3.88	2.85
Calcium, %	0.89	0.80	0.80	0.70	0.70
Phosphorous, %	0.79	0.71	0.63	0.68	0.62
Available phosphorous, %	0.59	0.40	0.40	0.32	0.33
SID Lysine, %	1.54	1.35	1.35	1.24	1.23
SID Threonine, %	0.97	0.84	0.80	0.80	0.76
SID Methionine, %	0.51	0.38	0.41	0.36	0.35
SID Tryptophan, %	0.27	0.27	0.28	0.27	0.27
**Analyzed Chemical Composition ^6^**					
Dry matter, %	90.10	89.00	87.00	91.30	87.40
Crude protein, %	22.70	24.70	17.30	23.00	17.70
Carbohydrate, % kcal	45.33	70.21	79.98	77.79	86.10
Crude fiber, %	1.30	2.00	1.60	3.50	2.10
Calcium, %	0.85	0.81	0.82	0.80	0.85
Phosphorus, %	0.75	0.65	0.56	0.70	0.61
Nitrogen, %	3.60	3.90	2.80	3.68	2.80

^1^ CON: standard protein diet; LP: low-protein diet, N1: nursery phase 1, fed for one week of study (from d 1 to 7); N2: nursery phase 2, fed for two weeks of study (from day 8 to 21; 7–11 kg body weight); N3: nursery phase 3, fed for three weeks of study (from day 22 to 42; 11–25 kg body weight). ^2^ Corn, fish meal, soybean meal, whey, lactose, corn starch, plasma spray-dried, soy protein concentrate, soybean oil, dicalcium phosphate, limestone, salt, and choline chloride (Cl) were obtained from Nutra Blend, LLC (Neosho, MO, USA). DL-methionine (99%) (MetAMINO^®^) and L-lysine, sulfate (Biolys^®^) were obtained from Evonik (Kennesaw, GA, USA). L-threonine (98.5%) and L-tryptophan (98%) were obtained from Ajinomoto (Overland Park, KS, USA). Chromium oxide was purchased from Fisher Scientific (Bartlesville, OK, USA). Selplex was obtained from Alltech (Lexington, KY, USA). ^3^ Vitamins premix was purchased from Nutra Blend, LLC (Neosho, MO, USA). Vitamins premix contained: vitamin A, 1,650,000 IU/kg; vitamin D3, 660,000 IU/kg; vitamin E, 17,600 IU/kg; vitamin B12, 13.2 mg/kg; vitamin K (menadione), 1320 mg/kg; niacin, 19,800 mg/kg; D-pantothenic acid, 11,000 mg/kg; riboflavin, 3300 mg/kg; phytase, 300,000 FYT/kg. ^4^ Trace minerals premix was purchased from Nutra Blend, LLC (Neosho, MO, USA). Trace minerals premix contained: iron, 73,000 ppm; zinc, 73,000 ppm; manganese, 22,000 ppm; copper, 11,000 ppm; iodine, 198 ppm; selenium, 198 ppm. ^5^ Values were calculated using National Swine Nutrition Guide (NSNG; V 2.0). ^6^ Diets were analyzed by ServiTech (Dodge City, KS, USA). The carbohydrate (% kcal of gross energy) was calculated from the estimated caloric value of carbohydrates at 4 kcal/g.

**Table 2:** Primer sequences, forward (F) and reverse (R), location on the template, amplicon size (bp), and GenBank accession numbers for both target and reference genes for reverse transcription-quantitative real-time polymerase chain reaction (RT-qPCR).

Genes ^1^	Sequence (5′ → 3′)	Location on Template	Amplicon Length (bp)	GenBank Accession Number
*SLC15A1*	F: AGCATCTTCTTCATCGTGGTCAA R: GTCTTGAACTTCCCCAGCCA	43–65 229–248	206	NM_214347.1
*SLC7A9*	F: ATCGGTCTGGCGTTTTAT R: GGATCTAGCACCCTGTCA	816–833 943–960	145	XM_021093176.1
*SLC7A8*	F: TTTCCAGGAACCTGACATCG R: ACATTGCAGTGACATAAGCG	576–595 756–775	200	XM_003128550.6
*SLC6A19*	F: CACAACAACTGCGAGAAGGA R: CCGTTGATAAGCGTCAGGAT	1101–1120 1236–1255	155	XM_003359855.4
*SLC3A1*	F: TTTCCGCAATCCTGATGTTC R: GGGTCTTATTCACTTGGGTC	1107–1126 1233–1252	146	NM_001123042.1
*SLC7A11*	F: CGGCTCCTGGGAAATTTCTC R: ACCATTCATGGAGCCAAAGC	1297–1316 1349–1368	72	XM_021101587.1
*SLC7A1*	F: TCTCATCCTAACGGGACTTTTAACTC R: GACCAGAACGTTGATACACGTGAA	2525–2550 2586–2609	85	XM_021065165.1
*β-Actin*	F: CTGCGGCATCCACGAAACT R: AGGGCCGTGATCTCCTTCTG	944–962 1071–1090	147	XM_021086047.1

^1^*SLC15A1*: solute carrier family 15 member 1 [40], *SLC7A9*: solute carrier family 7 member 9 [40], *SLC7A8:* solute carrier family 7 member 8 [40], *SLC6A19:* solute carrier family 6 member 19 [41], *SLC3A1:* solute carrier family 3 member 1 [41], *SLC7A11:* solute carrier family 7 member 11 [41], *SLC7A1:* solute carrier family 7 member 1 [42], and *β-actin:* beta-actin [43]. F: forward (sense) primer; R: reverse (antisense) primer.

**Table 3:** Growth performance and muscle fat and lean content of nursery pigs fed with two levels of dietary protein and three levels of a phytogenic water additive.

	Diets ^1^						SEM ^2^	*p*-Value		
Parameters	CON-NS	CON-LS	CON-HS	LP-NS	LP-LS	LP-HS		Protein	PWA ^3^	Protein × PWA
Initial BW ^4^, kg	8.82	9.10	9.63	9.05	8.76	8.76	0.17	0.36	0.78	0.46
Final BW ^4^, kg	25.34 *	26.08	29.09 *	24.89	25.00	23.25	0.56	0.02	0.72	0.09
ADG ^4^, kg/d	0.59	0.60	0.70 ^b^	0.57	0.58	0.52	0.01	0.01	0.75	0.07
ADFI ^4^, kg/d	0.86	0.95	0.97	0.84	0.91	0.86	0.02	0.23	0.30	0.69
ADWI ^4^, L/d	3.09	2.98	4.17	2.78	3.54 ^d^	2.98	0.17	0.35	0.31	0.12
G:F ^4^, kg/kg	0.69	0.63	0.72	0.67	0.62	0.61	0.01	0.01	0.97	0.72
G:P ^4^, kg/kg	2.97	2.83	3.09	3.83 ^c^	3.58	3.38	0.09	0.01	0.33	0.12
Muscle lean%	82.83	83.35	86.00	78.58	80.32	88.7 ^e^	0.86	0.01	0.01	0.01
Muscle fat%	16.62	16.06	13.51	20.85	13.33	9.41 ^e^	0.82	0.01	0.01	0.01

^1^ CON-NS: control diet with no phytogenic water additive (PWA) supplemented, CON-LS: control diet with a low dose of PWA (4 mL/L of water) supplemented, CON-HS: control diet with a high dose of PWA (8 mL/L of water) supplemented, LP-NS: low-protein diet with no PWA added, LP-LS: low-protein diet with a low dose of PWA (4 mL/L of water) added and LP-HS: low-protein diet with a high dose of PWA (8 mL/L of water) added. Values are the means. *n* = 8/diet. ^2^ SEM: standard error of the mean. ^3^ PWA: phytogenic water additive. ^4^ BW: body weight; ADG: average daily gain; ADFI: average daily feed intake; ADWI: average daily water intake; G:F: gain:feed ratio; G:P: gain:protein ratio. ^b^ *p* ≤ 0.05 CON-NS vs. CON-HS, ^c^ *p* ≤ 0.05 CON-NS vs. LP-NS, ^d^ *p* ≤ 0.05 LP-NS vs. LP-LS, and ^e^ *p* ≤ 0.05 LP-NS vs. LP-HS. * Within a row, values with a common superscript symbol tend to be different (0.05 < *p* ≤ 0.1).

**Table 4:** Weekly growth performance of nursery pigs fed with two levels of dietary protein and three levels of a phytogenic water additive.

	Diets ^1^						SEM ^2^	*p*-Value		
Parameters	CON-NS	CON-LS	CON-HS	LP-NS	LP-LS	LP-HS		Protein	PWA ^3^	Protein × PWA
**BWG ^4^, kg**										
Wk 1	1.98	2.32	2.97	1.36	1.24	2.77	0.12	0.04	0.47	0.18
Wk 2	4.05	4.24	4.20	3.33	4.13	3.22	0.13	0.03	0.19	0.41
Wk 3	5.01	4.80	4.91	4.96	5.09	3.90	0.18	0.39	0.47	0.20
Wk 4	5.48	5.88	7.37 ^b^	6.22	5.77	4.75	0.21	0.14	0.77	0.03
**CFI ^4^, kg**										
Wk1	3.35	3.63	3.76	2.97	3.10	3.82	0.14	0.32	0.19	0.69
Wk 2	5.11	5.83	6.17	4.61	5.33	5.80	0.19	0.21	0.04	0.98
Wk 3	7.30	7.72	7.38	6.79	7.83	6.85	0.22	0.49	0.34	0.80
Wk 4	8.28	8.92	9.67	9.33	9.49	7.86	0.25	0.89	0.72	0.05
**CPI ^4^, kg**										
Wk1	0.83	0.90	0.93	0.51	0.54	0.66	0.03	0.01	0.26	0.84
Wk 2	1.17 *^#^	1.34	1.41 ^#^	0.81 *^$^	0.94	1.02 ^$^	0.04	0.01	0.04	0.97
Wk 3	1.67 *	1.77	1.69	1.20 *	1.38	1.21	0.05	0.01	0.38	0.89
Wk 4	1.89	2.18	2.25	1.62	1.64	1.33	0.06	0.01	0.42	0.03
**CWI ^4^, L**										
Wk 1	12.31	15.95	14.45	12.42	10.90	13.95	0.92	0.34	0.73	0.46
Wk 2	24.19	23.18	25.02	12.54 ^c^	19.52	22.93 ^e^	1.50	0.05	0.30	0.36
Wk 3	25.51	22.90	30.71	26.09	29.98	23.65	1.55	0.96	0.93	0.20
Wk 4	30.04	26.43	34.94	26.13 *	38.39 *	24.72	1.85	0.81	0.63	0.04
**G:F ^4^, kg/kg**										
Wk 1	0.59	0.64	0.79	0.46	0.40	0.73	0.05	0.01	0.03	0.60
Wk 2	0.79	0.72	0.68	0.73	0.77	0.57	0.02	0.55	0.01	0.31
Wk 3	0.69	0.62	0.66	0.73	0.66	0.57	0.02	0.54	0.13	0.15
Wk 4	0.66	0.63	0.76	0.67	0.61	0.60	0.01	0.16	0.54	0.38
**G:P ^4^, kg/kg**										
Wk 1	2.39	2.58	3.19	2.67	2.30	4.20 ^e^	0.20	0.94	0.03	0.14
Wk 2	3.46	3.16	2.98	4.11	4.39	3.16	0.10	0.02	0.01	0.22
Wk 3	3.00	2.71	2.91	4.13 ^c^	3.69	3.22 ^e^	0.09	0.01	0.06	0.16
Wk 4	2.90	2.70	3.28	3.84 ^c^	3.52	3.57	0.07	0.01	0.56	0.27

^1^ CON-NS: control diet with no phytogenic water additive (PWA) supplemented, CON-LS: control diet with a low dose of PWA (4 mL/L of water) supplemented, CON-HS: control diet with a high dose of PWA (8 mL/L of water) supplemented, LP-NS: low-protein diet with no PWA added, LP-LS: low-protein diet with a low dose of PWA (4 mL/L of water) added and LP-HS: low-protein diet with a high dose of PWA (8 mL/L of water) added. The *p*-values for the overall model effect of protein, PWA, week, protein × PWA, protein × week, PWA × week and protein × PWA ×week for BWG were 0.01, 0.61, 0.01,0.07, 0.85, 0.48, and 0.12, for CFI were 0.35, 0.31, 0.01, 0.68, 0.86, 0.19, and 0.05, for CPI were 1.00, 0.94, 0.01, 0.01, 0.09, 0.11, and 0.01, for CWI were 0.26, 0.18, 0.01, 0.20, 0.39, 0.57and 0.06, for G:F were 0.05, 0.87, 0.26, 0.42, 0.01, 0.01 and 0.57, and for G:P were 0.66, 0.52, 0.01, 0.07, 0.42, 0.01 and 0.01, respectively. Values are the means. *n* = 8/diet. ^2^ SEM: standard error of the mean. ^3^ PWA: phytogenic water additive. ^4^ BWG: body weight gain (weekly); CFI: cumulative feed intake; CPI: cumulative protein intake CWI: cumulative water intake; G:F: gain: feed; G:P: gain: protein. ^b^ *p* ≤ 0.05 CON-NS vs. CON-HS, ^c^ *p* ≤ 0.05 CON-NS vs. LP-NS, and ^e^ *p* ≤ 0.05 LP-NS vs. LP-HS. *^#$^ Within a row, values with a common superscript symbol tend to be different (0.05 < *p* ≤ 0.1).

**Table 5:** The concentration (nmol/mL) of plasma nitrogen-containing compounds of nursery pigs fed with two levels of dietary protein and three levels of a phytogenic water additive.

Item	Diets ^1^						SEM ^2^	*p*-Value		
	CON-NS	CON-LS	CON-HS	LP-NS	LP-LS	LP-HS		Protein	PWA	Protein × PWA
Valine	343.7	372.7	518.0 ^b^	130.1 ^c^	246.6 ^d^	86.5	26.3	0.01	0.01	0.01
Methionine	41.2 *	59.8 *	68.6	64.5	72.3	73.0	3.2	0.02	0.04	0.41
Threonine	328.3 ^$^*	388.4	460.0 ^$^	631.5 *	660.2	590.7	28.9	0.01	0.10	0.26
Isoleucine	171.9	190.6	301.3 ^b^	100.4 ^c#^	121.7 ^#^	75.9	13.4	0.01	0.01	0.01
Leucine	268.9	314.1 ^a^	428.6 ^b^	255.9 ^#^	294.7 ^#^	214.3	12.9	0.01	0.01	0.01
Phenylalanine	126.4 *	146.2	194.7 ^b^	100.8 *^#^	118.8 ^#^	89.0	6.6	0.01	0.01	0.01
Tryptophan	55.8	70.4	109.4 ^b^	81.2	93.7	112.8	4.9	0.02	0.01	0.42
Lysine	189.6 *	267.6 *	353.6 ^b^	529.0 ^c^*	618.3	365.7 *	29.0	0.01	0.09	0.01
Histidine	136.3	137.3	182.2 ^b^	74.2 ^c^	95.3 ^d^	50.4 ^e^	8.0	0.01	0.28	0.01
Arginine	39.7	105.2	393.8 ^b^	63.8	79.4	124.6	11.9	0.01	0.01	0.01
Aspartic acid	79.2	76.7	41.67 ^b^	58.3	78.6	30.17	4.6	0.16	0.01	0.42
Serine	307.4	382.4 ^a^	401.3 ^b^	395.7 ^c^	420.5	285.6 ^e^	11.9	0.84	0.02	0.01
Glutamic acid	444.0	485.8	261.6 ^b^	499.9	594.9	330.3 ^e^	24.9	0.03	0.01	0.81
Glutamine	632.8	683.9	840.5	770.4	892.4	862.8	29.9	0.06	0.05	0.64
Glycine	1420.1	1772.4 ^a^	1701.3 ^b^	2168.0 ^c^	2027.7	1887.3	54.5	0.01	0.45	0.01
Alanine	778.4	917.4 ^a^	989.1 ^b^	1054.4 ^c^	1262.6 ^d^	915.5	34.0	0.01	0.01	0.01
Tyrosine	167.3	199.6	305.1 ^b^	159.2	181.9	129.9	10.5	0.01	0.03	0.01
Asparagine	169.3	208.7	291.9 ^b^	164.0	181.4	150.1	11.9	0.01	0.10	0.02
Proline	457.2	528.6 ^a^	676.9 ^b^	505.6 ^#^	538.3	396.8 ^#^	17.6	0.01	0.07	0.01
Ammonia	367.3	452.7	245.3	403.1	424.0	232.1 ^e^	19.1	0.62	0.01	0.25
Creatinine	115.6	131.1	161.7	112.9	129.8	81.7	6.2	0.01	0.43	0.01
Taurine	266.7	281.0	141.4 ^b^	273.8	357.3	96.4 ^e^	19.1	0.27	0.01	0.01
Sarcosine	50.7	66.1 ^a^	62.7 ^b^	73.6 ^c^	80.9	74.0	2.2	0.01	0.02	0.31
3,methylhistidine	10.9	10.2	12.5	6.71 ^c^	7.6	6.8	0.4	0.01	0.42	0.08
1,methylhistidine	58.4	70.3	67.3	56.7	57.9	60.2	3.0	0.26	0.63	0.78
Ethanolamine	38.1	47.1	25.0	29.4	20.3	17.9	3.0	0.01	0.01	0.26
Carnosine	31.9	29.7	30.4	22.7	23.1	19.2	1.7	0.01	0.81	0.84
Hydroxylysine	11.5	14.6	14.1	9.6	9.8	12.5	0.5	0.01	0.03	0.24
Citrulline	74.5	89.6	96.4	70.1	75.8	79.1	2.7	0.02	0.04	0.53
Ornithine	352.8 ^$^	360.5	285.5 ^$^	251.5 ^#^	311.4 ^#^	125.2	17.2	0.01	0.01	0.16
α-aminobutyric acid	35.6	41.0	13.4	39.6	34.0	17.7	3.1	0.93	0.01	0.63

^1^ CON-NS: control diet with no phytogenic water additive (PWA) supplemented, CON-LS: control diet with a low dose of PWA (4 mL/L of water) supplemented, CON-HS: control diet with a high dose of PWA (8 mL/L of water) supplemented, LP-NS: low-protein diet with no PWA added, LP-LS: low-protein diet with a low dose of PWA (4 mL/L of water) added and LP-HS: low-protein diet with a high dose of PWA (8 mL/L of water) added. Values are the means. *n* = 6/diet. ^2^ SEM: Standard error of the mean. ^a^ *p* ≤ 0.05 CON-NS vs. CON-LS, ^b^ *p* ≤ 0.05 CON-NS vs. CON-HS, ^c^ *p* ≤ 0.05 CON-NS vs. LP-NS, ^d^ *p* ≤ 0.05 LP-NS vs. LP-LS, and ^e^ *p* ≤ 0.05 LP-NS vs. LP-HS. *^#$^ Within a row, values with a common superscript symbol tend to be different (0.05 < *p* ≤ 0.1).

## Data Availability

All relevant data is listed in the manuscript.

## Supplementary Materials

The following are available online at https://www.mdpi.com/2076-2615/11/2/555/s1, Table S1: Ingredients and chemical composition of the phytogenic water additive used in this study; Figure S1: Body weight and feed intake of nursery pigs fed with two levels of dietary protein and three levels of a phytogenic water additive; Figure S2: Serum total antioxidant capacity of nursery pigs fed with two levels of dietary protein and three levels of a phytogenic water additive.
