# Supplementary material for: Effect of a Phytogenic Water Additive on Growth Performance, Blood Metabolites and Gene Expression of Amino Acid Transporters in Nursery Pigs Fed with Low-Protein/High-Carbohydrate Diets
Source: Animals (Basel). 2021 Feb 20;11(2):555. doi: 10.3390/ani11020555 (PMC7923792; doi:10.3390/ani11020555)
Supplement: Supplementary file 1 [file animals-11-00555-s001.pdf]

Supplementary Tables

Table S1. Ingredients and chemical composition of the phytogenic water additive (Herbanimals®) used in this study (as-fed basis)

Items	Herbanimals®
**Ingredients, %**	
Pandanus amaryllifolius Roxb	4.83
Phyllanthus niruri	24.15
Amomum cardamomum	4.83
Zingiber zerumbet	13.04
Apium Graveolens	14.49
Anethum Graveolens	14.49
Ocimum americanum	4.83
Cinnamomum burmannii Blume	4.83
Myristica fragrans Houtt	4.83
Zingiber officinale roscoe	9.66
**Analyzed Chemical Composition, mg/100 g**	
Calcium	4.99
Magnesium	4.16
Iron	1.01
Vitamin B6	0.46
Vitamin B12	0.69
Vitamin E	0.86
Vitamin B1	0.04
Vitamin B2	0.19
Arginine	15.58
Vitamin B3	0.14
Aspartic acid	10.45
Pantothenic acid	1.39

Supplementary Figures

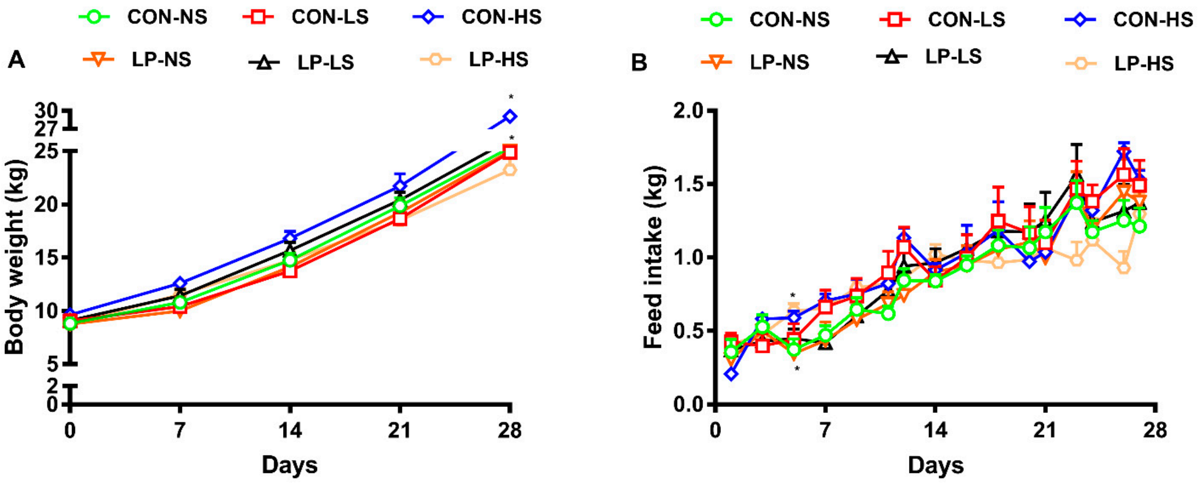

Figure S1. Body weight (A) and feed intake (B) of nursery pigs fed with two levels of dietary protein and three levels of a phytogenic water additive (PWA). The *p* values for the overall model effect of protein, PWA, time, protein × PWA, protein × time, PWA × time and protein × PWA× time for body weight were 0.05, 0,49, 0.01, 0.49, 0.01, 0.05 and 0.01 and for feed intake were 0.07, 0.10, 0.01, 0.37, 0.68, 0.47 and 0.31, respectively. CON-NS: control diet with no PWA supplemented, CON-LS: control diet with a low dose of PWA (4 ml/L of water) supplemented, CON-HS: control diet with a high dose of PWA (8 ml/L of water) supplemented, LP-NS: low protein diet with no PWA added, LP-LS: low protein diet with a low dose of PWA (4 ml/L of water) added and LP-HS: low protein diet with a high dose of PWA (8 ml/L of water) added. [*]Among groups, values with a common superscript symbol tended to be different ($0.05 < p \leq 0.1$). Values are means ± SEM. *n*=8/dietary group.

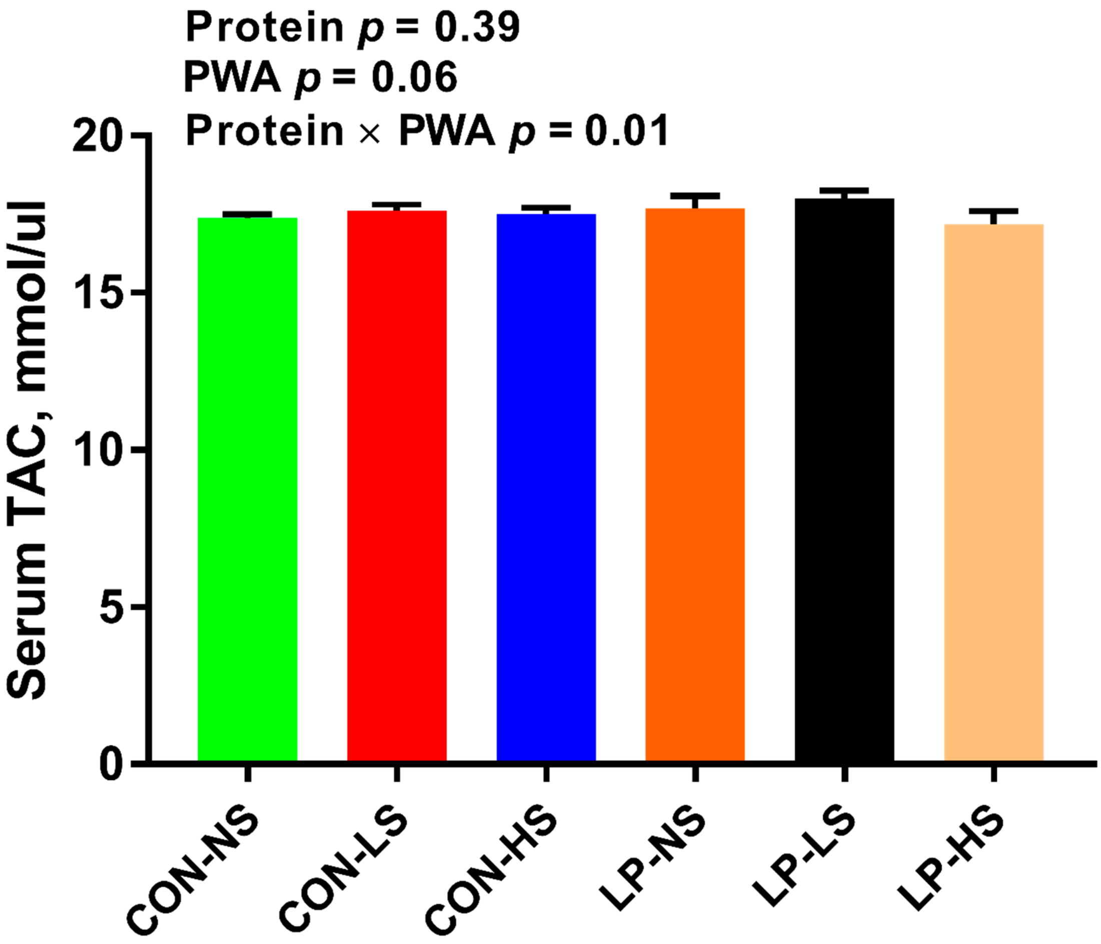

Figure S2. Serum total antioxidant capacity of nursery pigs fed with two levels of dietary protein and three levels of a phytogenic water additive (PWA). CON-NS: control diet with no PWA supplemented, CON-LS: control diet with a low dose of PWA (4 ml/L of water) supplemented, CON-HS: control diet with a high dose of PWA (8 ml/L of water) supplemented, LP-NS: low protein diet with no PWA added, LP-LS: low protein diet with a low dose of PWA (4 ml/L of water) added and LP-HS: low protein diet with a high dose of PWA (8 ml/L of water) added. Values are means ± SEM. *n*=8/dietary group.
